# Frame fit: safety, acceptability and effectiveness of a community-based fall prevention exercise intervention for walking frame users, a randomised controlled trial

**DOI:** 10.1186/s12877-026-07293-1

**Published:** 2026-04-16

**Authors:** Julie Whitney, Matthew DL O’Connell, Stephen HD Jackson, Finbarr C Martin, Ben Carter

**Affiliations:** 1 The School of Life Course & Population Sciences, Faculty of Life Sciences and Medicine, King’s College London, SE1 1UL London, UK; 2 Department of Clinical Gerontology, King’s College Hospital, Denmark Hill, London, SE5 9RS UK; 3 Department of Biostatistics and Health Informatics, Institute of Psychiatry, Psychology and Neuroscience. King’s College London, SE5 8AF London, UK

**Keywords:** Walking aid, falls, exercise, balance, frailty

## Abstract

**Background:**

Walking frame users are at increased risk of falls due to gait and balance impairments and restrictions caused by the frame itself. While exercise is an effective fall prevention intervention in the general population, there are no programmes specific to walking frame users. Adaptations may be required for walking frame users to achieve an appropriate intensity and dose of fall prevention exercise without increasing the propensity to fall while exercising. The Frame Fit intervention is a home exercise programme adapted for the needs of frame users. The aim of this study was to evaluate safety, acceptability and effectiveness of the Frame Fit intervention.

**Methods:**

Community dwelling older people (aged ≥ 65) who use a walking frame were recruited to the randomised controlled trial and allocated to the 6-month Frame Fit intervention, a programme of home exercises prescribed and progressed by a physiotherapist, or to usual care. Falls (primary outcome) were recorded using diaries for 12 months from randomisation. Physical performance (grip strength, balance, gait speed and sit-to-stand), timed up and go, physical activity, fear of falling and health-related quality of life was measured at baseline and after six months. Secondary healthcare use and mortality was collected from electronic records for 12 months from randomisation.

**Results:**

Of 117 participants randomised, (59 to intervention, 58 to usual care), 86 were followed up at six months and 83 provided 12 month falls data. There were two intervention-related adverse events. There were more falls reported by the intervention group than control group at six months (adj, IRR: 2.10, 95%CI 1.06–3.98) but no difference in falls at 12 months (adj.IRR:1.76, 95%CI 0.93–3.33). Adherence was satisfactory but there was low uptake to the trial. There were no differences found in the secondary outcome measures.

**Conclusions:**

Frame Fit intervention offered a satisfactory safety profile, was acceptable to those who participated and feasible. Due to low recruitment rates, the study was underpowered to detect a difference in falls or other outcomes. Further research is needed to optimally tailor fall prevention interventions to walking frame users.

**Trial registration:**

The protocol was prospectively registered as a clinical trial with the ISRCTN (clinical trial number: 57645734) on 11/09/2014.

**Supplementary Information:**

The online version contains supplementary material available at 10.1186/s12877-026-07293-1.

## Background

Walking aids are frequently recommended with the intention of improving walking stability and reducing the risk of falls. Walking aids range in the degree of stability offered, from a single stick to a walking frame. For this study, a walking frame is defined as ‘a walking aid with three or four points of ground contact that is operated with both arms with ground contact being ferrule tipped, wheel-tipped or a combination of the two’. Walking frames reduce load bearing through the lower limbs [1], with the potential to decrease joint pain, with wheeled versions improving exercise tolerance [2]. Any walking aid increases the base of support improving stability and enhancing sensory feedback [3].

A study of people aged > 65 in the United States estimated that 24% used any category of walking aid and 11% specifically a ‘walker’ [4]. Similar prevalence data was found in a Canadian study where 19% of people aged over 70 reported using walking sticks and 14% using a frame [5]. Frailer older people are even more likely to use walking frames and in previous work we found that 40% of care home residents required a frame to walk [6].

Being a walking aid user is associated with increased fall risk [7, 8]. In a cohort study investigating fall risk factors in care homes, we found walking frame use to be predictive of falls, independent of balance ability [6]. Using a walking frame may alter postural responses. Muscle activity is task specific and plastic, in the sense that neuromuscular responses will adapt to the situations they are exposed to. Therefore, using a walking frame over time could lead to inadequate lower limb postural responses to perturbations. Using a frame also hinders negotiation of obstacles, changes the way a person moves and requires additional attention [9]. There is also evidence to suggest that the frame itself could cause falls, acting as a physical barrier to appropriate stepping or upper limb saving reactions, and increasing the risk of fall-related injury [3]. One study found that frame users had seven times the rate of fall-related injuries than those who walked with a stick [10]. Thies et al. found that narrow frames provided significantly less stability than those of average width, and stability improved as the amount of body weight transfer onto the frame increased [11].

Exercise that includes frequent and regular highly-challenging balance training effectively reduces risk of falls [12] and has the potential to slow the development of frailty [13]. However, there is a theoretical risk that in inactive older adults living with frailty, starting a new exercise programme could increase falls due to increased exposure to falling as a result of spending more time standing and walking. Use of a walking aid is a common exclusion criterion for exercise trials [14–16]. A review of inclusion / exclusion criteria from studies in the Cochrane review of exercise for prevention of falls [17], revealed that > 20 trials excluded participants based on walking frame use. Only two studies were identified that recruited participants because they used a walking aid [18, 19]. No fall prevention exercise trial has specifically focused on walking frame use or those who regularly use them.

Considering the prevalence of frame use and the associated fall risk, effective methods to prevent falls tailored to this population are required. The aim of this study was to evaluate the feasibility, safety, acceptability and effectiveness of a fall prevention exercise programme specifically designed for frame users; ‘Frame Fit’.

The objectives were:

1. To test the safety profile of the Frame Fit intervention.
2. To determine the acceptability and feasibility of Frame Fit by measuring uptake, exercise adherence, number and reason for dropouts, engagement in exercise sessions and intervention fidelity.
3. To determine the clinical effectiveness of Frame Fit on primary and secondary outcomes.

## Methods

### Study design

A multicentre single blind randomised controlled trial comparing Frame Fit versus usual care.

### Setting

This study was undertaken in a socioeconomically diverse inner-city population in London (UK). The processes of gaining consent, baseline and follow up measurement and delivery of the intervention were all undertaken in the participants’ homes.

### Recruitment

Recruitment methods aimed to identify as many local residents as possible who would be eligible to participate in the study.

Participants were recruited from one acute general university-affiliated hospital, one neighbouring community health service, sheltered accommodation units and primary care practices (GPs). As the intervention was delivered by one site team, only local recruitment sites were approached. From the acute hospital, potential participants were approached on inpatient wards prior to discharge and in outpatient clinics. Wards and clinic lists were screened daily by a study research nurse. Physiotherapists undertaking domiciliary visits in the community trust aligned with the participating acute hospital were provided with information about the study and asked to identify and consent eligible participants. GP practices in Southeast London were approached through the regional Clinical Research Network and 13 GP practices participated. Each practice used standardised records searching (search terms used ‘walking frame’, ‘poor mobility’, ‘frailty’, ‘housebound’ and ‘aged over 65’ followed by GP review of those selected to ensure suitability) to identify potential participants who were sent a mailout about the study. Local sheltered accommodation units were identified via local authority lists and managers contacted. Three sheltered accommodation units agreed to researchers holding an information session about the research in their day room to invite potential participants.

### Participants

Participants were eligible for the study if they were a regular walking frame user and aged ≥ 65. Regular frame use was defined as ‘using the frame for the majority of walking and standing tasks on a daily basis’. There was no minimum duration of walking frame use stipulated. All participants had to have completed physiotherapy or rehabilitation programmes (if relevant) prior to enrolment in the study.

Safety to participate was ascertained based on cognitive and physical performance. Those with cognitive impairment or a diagnosis of dementia were not excluded but to ensure safe and effective participation, those with MMSE [20] < 24/30 or a diagnosis of dementia had to be living with someone who would be able to support the home exercise programme and complete falls diaries and all participants had to be able to follow simple movement instructions at the baseline assessment. For the same reasons, those with insufficient English language to provide informed consent and follow study procedures were also excluded. Participants with a recent history of delirium were eligible if 6 weeks had passed since the onset of delirium to ensure stability.

To ensure physical function was adequate for safe unsupervised home exercise, participants had to be able to walk 6 m without resting, be able to rise from a chair without human assistance and have no restrictions on weightbearing. No a-priori criteria were made based on the suitability of the home environment and no participants were excluded due to an unsafe home environment. Finally, potential participants were excluded if their clinician (hospital doctor or GP) considered them unlikely to survive more than six months.

### Baseline visit

During the baseline visit, data including demographics, medical conditions, medication use, function (Barthel index [21]) and cognition (ACE-R [22]) were collected. The Fried Frailty Index [23] was calculated from baseline information collected about self-reported weight loss and fatigue, slowness (Timed up and Go > 19 s), weakness (grip strength < 20 kg for females and < 30 kg for males), and physical activity (1SD below IPEQ-W normative data presented by Debleare et al.) [24]. Participants who, upon baseline assessment were found not to meet the inclusion/exclusion criteria were not randomised.

### Randomisation

The randomisation sequence allocation was generated using varying permuted blocks (block size 2 and 4) stratified by gait speed (< 0.6 m/s) by King’s Clinical Trials Unit (KCTU) and stored on an online web-based server. Access was centrally controlled and concealed by KCTU and shared with the intervention physiotherapist to implement allocation.

### The frame fit intervention

The intervention was designed to address mobility and balance challenges experienced by walking frame users. Before starting the programme, each participant’s frame was checked for damage and ferrule wear and appropriate maintenance carried out.

Qualified physiotherapists with experience of between 2 and 15 years delivered the intervention to individual participants in their own homes. Supervision of the physiotherapy team was provided by an experienced physiotherapist (the lead author). The exercises were adapted from the Otago exercise programme (OEP) [25] which is effective at reducing falls in community dwelling older people.

Exercises were prescribed and progressed during physiotherapy home visits and participants were encouraged to do the whole exercise programme at least three times a week. The full programme consisted of five warm-up, five strengthening, 11 balance, and two cool-down exercises which were selected and tailored by the physiotherapist (see Table 1). The aim was to prescribe as many of the exercises in the programme as possible with exercises omitted if a participant was unable to perform safely. Where participants did not have the endurance to complete the full exercise set, the therapist would prioritise which exercises to prescribe based on individual baseline assessment findings, ensuring a mix of strength and balance exercises were included and with the aim of adding further exercises at progression visits. Strength exercises were prescribed at a moderate intensity. Balance training, defined as ‘exercise that involves reducing the base of support or moving the centre of gravity while minimising the use of upper limbs for support’ [12] was prescribed at high intensity, where upper limb support is modulated to make the person ’somewhat wobbly’ but not to the extent this would cause a fall. If not using any upper limb support, participants were instructed to remain near a source of support to use if needed. Twice weekly outdoor walking for up to 30 min, either accompanied or unaccompanied was encouraged if the participant was not housebound and had a suitable outdoor walking frame.

**Table 1:** Exercises in the programme

Exercise	Description	Progression	Adaptation from original OEP
Warm up exercises:			
1. Neck rotations	Sitting in chair 5x looking side to side	-	Sitting only
2. Neck retraction	Sitting in chair 5x tucking chin in	-	Sitting only
3. Trunk rotation	Standing, rotate side to side 5x	With / without holding	Frame for support if needed
4. Trunk extension	Standing, extend lower back 5x	With / without holding	Frame for support if needed
5. Ankle dorsiflexion and plantarflexion	Sitting, knee extended, 10x	-	Sitting only
Strength:			
1. Squats	10x	1 or no hands, 2 sets	Frame for support if needed
2. Heel raises	10x	1 or no hands, 2 sets	Frame for support if needed
3. Toe raises	10x	1 or no hands, 2 sets	Frame for support if needed
4. Knee extension	10x with 2Kg weight (sitting)	↑ weight and reps	No difference
5. Hip abduction	10x with 2Kg weight (standing)	↑ weight and reps	Frame for support if needed
Balance:			
*Walking and manoeuvring*:			
1. Side stepping with frame	Step 5 steps to the right and 5 to the left 4x	8 repetitions	Frame for support and shorter distance.
2. Stepping forward and backwards with frame	Step forwards then backwards 10 steps 2x	4 sets	Instead of backwards walking with worktop
3. Walking and turning with the frame	Walk and turn in figure of eight 2x	2 sets	With frame in instruction and illustration.
*Transfers*:			
4. Sit to stand	6 times, 2 sets (correct hand placement)	3–4 sets (reduce use of UL)	Frame in front in illustration and correct placement of hands in instructions.
5. Step around transfer	Stand up, walk to nearby chair / bed and sit down 4x	6x (transfers without frame)	Added exercise specific to frame users
*Standing balance*:			
6. Feet close together	Stand 10 s	With / without holding	Added exercise specific to frame users (with expectation of lower levels of balance skill)
7. Reaching	Standing, reaching sideways and forwards	With / without holding	As above
8. Bending down	Standing, reaching towards feet	With / without holding	As above
9. Turning	Standing turning to reach behind	With / without holding	As above
10. Tandem standing	Tandem standing	With / without holding	Frame for support if needed
11. Side stepping without the frame	Side stepping next to kitchen worktop 5x	1 or no hands	Same as original OEP
Cool down:			
1. Calf stretch	Standing hold 10 s		Additional stretch
2. Hamstring stretch	Sitting leg outstretched, hold 10 s		Same as original OEP

**Fig. 1:**
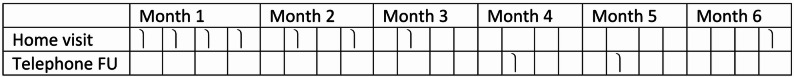
Schedule of exercise visits and telephone follow up

**Fig. 2:**
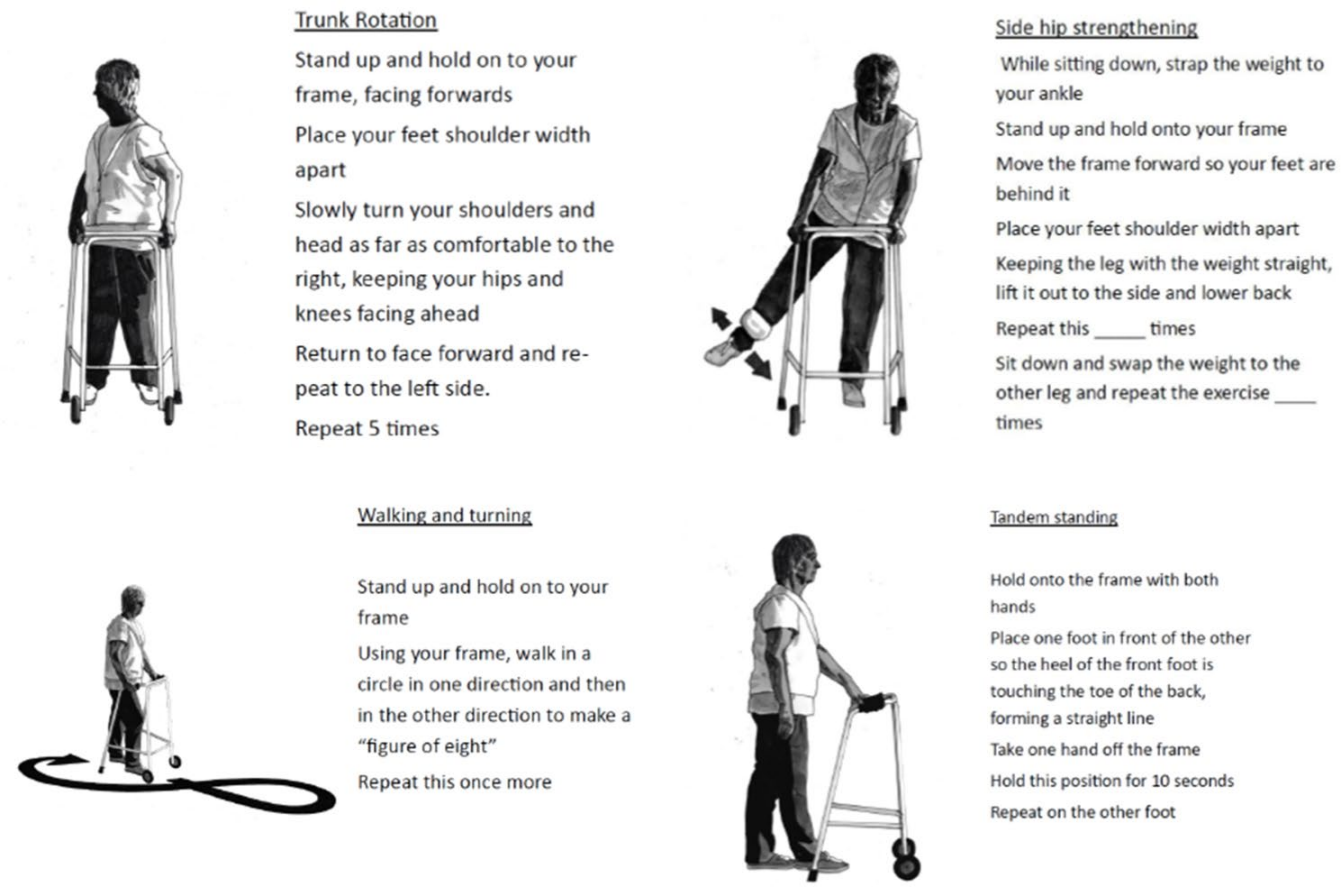
Examples of exercise images from the manual

There were eight scheduled home visits and two telephone follow ups over the course of the six-month intervention (see Fig. 1 for visit schedule). This is twice as many visits as the original OEP as it was anticipated that participants would require more support to successfully progress exercises and maintain adherence due to their likely higher prevalence of frailty. An exercise manual was given to each participant which included information about using the frame safely and what to expect when exercising. Pictures and descriptions of each of the exercises, at each gradation, were available as A4 pages which could be selected and added to the exercise manual (see Fig. 2 for examples).

Exercise instructions from the original OEP were adapted to include using the frame for support, illustrations redesigned to include a frame and additional functional balance exercise related to the needs of frame users included. Table 1 indicates how each exercise was adapted from the original OEP. No formal feasibility evaluation was done prior to the trial but all exercises had been used before in clinical care and the exercise manual was reviewed by the study patient involvement group.

The TIDieR checklist for the intervention description can be found in the supplementary data [27].

### Usual care

Participants in the usual care group received written information about using their frame safely and their frame was checked for damage and ferrule wear and appropriate maintenance carried out at the baseline visit. No other change in their care was initiated.

### Data collection

#### Blinding of personnel

The participants and treating physiotherapists were not blinded. Blinded assessors (trained nurses and physiotherapists) undertook all baseline visits and completed outcome data collection. Blinded assessors confirmed they did not know the allocation prior to each assessment timepoint.

#### Safety profile

Adverse events (AEs) were collected for the duration of the study, reported by the treating physiotherapists or the participant at an exercise session or by use of the falls diary. Secondary care use during the 12-month follow-up (emergency department attendance, hospital admission and length of stay) were collected, and survival status (dead/alive) via local electronic health records for 12 months after randomisation.

#### Acceptability and feasibility

Dropout numbers and reasons were recorded as well as the number of physiotherapy sessions completed. At each physiotherapy session, engagement was rated by the treating physiotherapist using a visual analogue scale, where 0 = no engagement and 10 = full engagement. Exercise prescriptions were recorded for each physiotherapist visit. Each participant was given an exercise diary to record home exercise.

#### Clinical effectiveness

The primary outcome measure was falls defined as ‘an unexpected event in which the person comes to rest on the ground, floor or lower level’ [28] measured for 12 months following randomisation. Falls were self-reported by participants using a diary which was returned monthly in a stamped addressed envelope and required participants to indicate if they had fallen or not for each day and any injuries sustained. If a fall was reported or the diary not returned, the participant was contacted by telephone. More details on reported falls collected over the telephone included location of fall, suspected cause and treatment given.

Secondary outcome measures were designed to evaluate factors which influence fall-risk to better understand the mechanism by which the intervention may act. All assessments were undertaken in the participants’ homes and repeated at baseline and after 6 months. Balance (i.e. ability to stand with feet together, near tandem, tandem or single leg), strength (sit-to-stand ability) and gait speed were measured as per the Short Physical Performance Battery [29]. Other measurements included the Timed Up and Go [30], grip strength using a manual Jamar dynamometer, physical activity (Incidental and Planned Activity Questionnaire for older people (IPEQ) [24]) and concern about falling (Short-Form Iconographical Falls Efficacy Scale International (FES-I) [31]). Health related quality of life was measured using the EQ5D [32].

#### Sample size calculation

A sample size calculation (using 80% power and 5% significance levels) used data from similar study designs [33, 34]. To reduce rate of falls from 0.5 per participant in one year to 0.3 (IRR = 0.60) allowing for a 15% dropout rate would require a total sample size of 240. This would also have at least 80% power to detect improvements of 12 s in the standing balance scale (baseline 17, SD 13) and improvement of 10 s to complete the Timed Up and Go (baseline 22 s, SD 12).

#### Data analysis

Descriptive statistics for AEs and healthcare use were presented by trial arm and intervention-related AEs described. Time to mortality was presented using Kaplan-Meier survival curves and analysed using a Cox regression adjusted by age and sex and summarised with an adjusted Hazard Ratio (aHR) presented with 95% confidence intervals (CI).

Dropouts were counted, the mean percentage of sessions completed, and mean session engagement calculated. Mean number of exercises prescribed over the 8 sessions were presented by exercise type.

Reasons for missing data were coded by the assessor. If a participant was physically/cognitively unable to undertake a test or was not followed up due to a deterioration in function or death, imputation of the worst score recorded in the dataset was applied (detail of missing data in Table 2).

**Table 2:** Handling of missing data

		Reason for missing data			
	*N* (%)	Unable to complete^1^	Dropped out / died^2^	Other^3^	Data imputed used (see key below)
BASELINE					
FES-I	3	-	-	3	N/a
IPEQ	3	-	-	3	N/a
Balance score	0	-	-	-	N/a
Gait speed	0	-	-	-	N/a
Timed up and go	0	-	-	-	N/a
Grip strength	0	-	-	-	N/a
Equation 5D	0	-	-	-	N/a
FOLLOW UP					
FES-I	43	10	7	26	40
IPEQ	43	10	7	26	0
Balance score	29	7	7	15	0 s
Gait speed	56	10	7	39	0 m/sec
Timed up and go	57	10	7	40	420 s
Grip strength	53	10	7	36	6 kg
Equation 5D	42	10	7	25	-0.172

^1^Given this code if unable to complete the assessment due to physical, functional or cognitive impairments. Worst score from dataset imputed to manage missing data

^2^ Given this code if dropped out due to health deterioration, being unable to stand up or died. Other reasons for drop out were coded as missing at random. Worst score from dataset imputed to manage missing data

^3^Given this code if reason for missingness not known, or drop out for reason other than health. No imputation applied to these data

The incidence rate ratio (IRR) for falls between the two arms at 6 and 12-month follow up was analysed using negative binomial regression analysis adjusted for age and sex and the proportion of fallers in each arm summarised using relative risk with 95% CIs. For the secondary outcome measures, continuous outcomes were analysed using linear regression analysis adjusted for age, sex and baseline score and presented as adjusted Odds Ratios with 95% CIs. EQ5D scores were calculated from raw data using the English (ENG) EQ-5D-5 L Devlin value set [35].

Data from the Balance test and Timed Up and Go (dichotomised into ≥ 30 and < 30 s) were categorized into improved, no change or worse between baseline and follow up by trial arm. Finally, a ratio of falls to physical activity scores was calculated for fallers (follow up IPEQ score / falls recorded in the first 6 months of follow up) and compared between arms.

An intention-to-treat approach was used for all analyses with significance set at *p* < 0.05.

Ethics approval was granted by the Camberwell and St Giles Ethics Committee, NHS Health Research Authority, United Kingdom (REC ref: 14/LO/0556) and the protocol was prospectively registered with the ISRCTN (clinical trial number: 57645734).

**Fig. 3:**
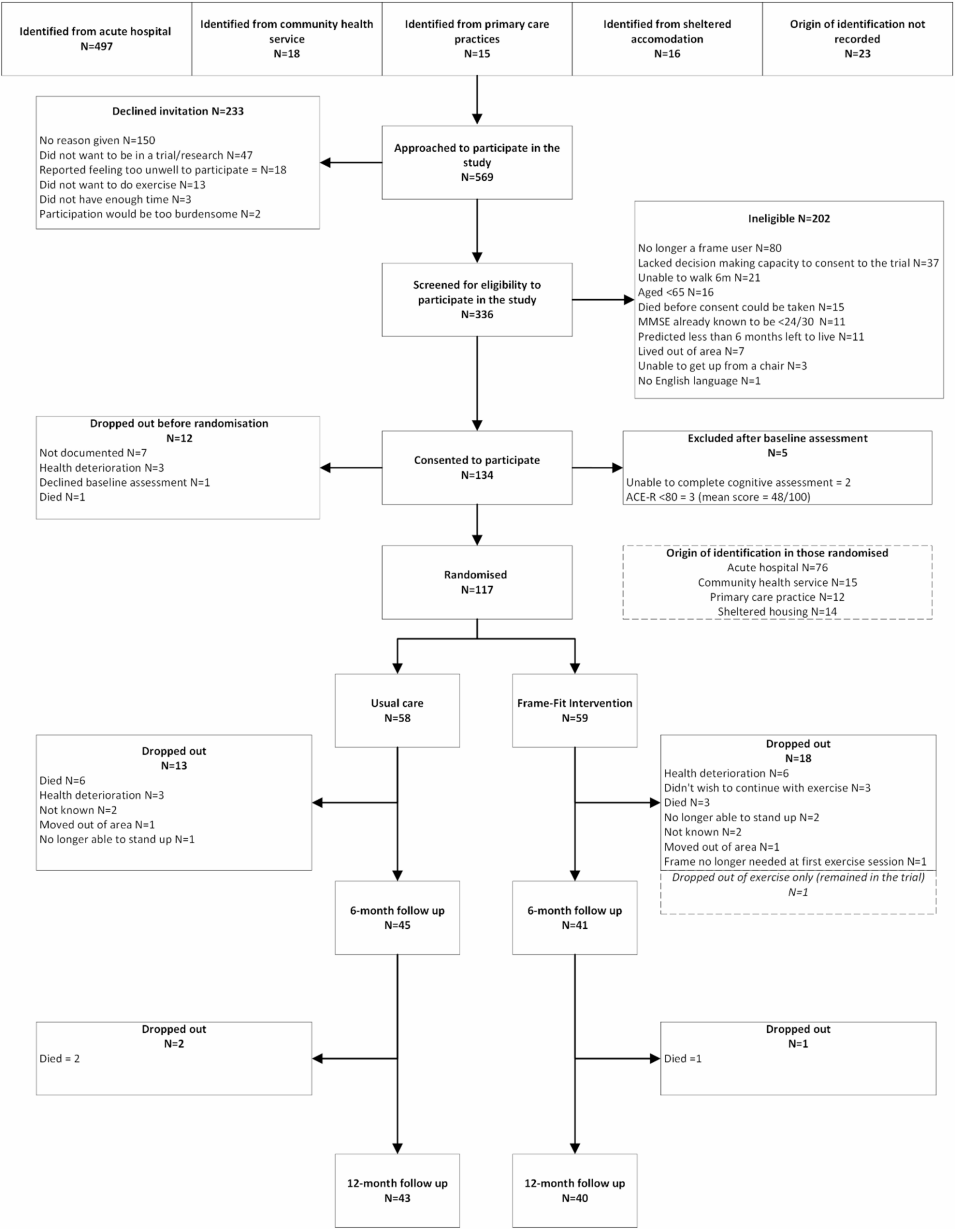
Consort diagram for participant flow

## Results

Five hundred and sixty-nine people were approached about participating in the study with 233 declining, 202 being ineligible and 134 participants consenting to participate. Twelve participants dropped out before randomisation and five were not randomised due to not meeting inclusion criteria upon baseline assessment. All five were excluded due to impaired cognition, two were unable to undertake the ACE-R and three demonstrated significant cognitive impairment and did not have a someone to support them with the home exercise/diaries. One hundred and seventeen participants were randomised with 59 allocated to the Frame Fit intervention and 58 to usual care. Eighty-six participants completed the 6-month follow up assessment and 83 provided 12-month falls follow up (see Fig. 3 for study Consort diagram). The first participant was randomised in January 2015 and the final 12-month fall follow up was completed in April 2019.

The mean age of participants was 83.4 (SD 7.7) ranging between 65 and 98 and 85 (72%) were female. The majority (*N* = 79, 68%) of participants were classified as frail using the Fried frailty phenotype. Participants were prescribed a mean of 8 medications (range 1–28) and had a mean of 5 conditions (range 0–11) with the most common being hypertension (59%), osteoarthritis (OA) (47%), diabetes (34%), heart failure (19%) and stroke (17%). While 8 participants had a dementia diagnosis, 84 (72%) demonstrated evidence of cognitive impairment with a baseline ACE-*R* < 80, the mean ACE-R being 69.6 (SD 15.3).

The usual care group were older and more likely to be frail and have cognitive impairment with no other differences between groups at baseline as indicated in Table 3. Data on unblinding at follow up were available for 28 participants with 8 (29%) cases of unblinding.

**Table 3:** Baseline data for randomised participants

	Usual care			Frame Fit Intervention		
	Total	Number	Percentage	Total	Number	Percentage
Demographics						
Female	58	44	76%	59	41	69%
Ethnicity	57			59		
White		45	79%		35	59%
Black		10	18%		20	34%
Asian		1	2%		2	3%
Mixed		0	-		0	-
Other		1	2%		2	3%
Walking aid used at baseline:	58			57		
Delta frame		1	2%		2	4%
Rollator frame		29	50%		29	50%
Trolley		4	7%		4	7%
Zimmer frame		24	41%		22	39%
Frailty (Fried)*	58			59		
0		0	0%		2	3.4%
1		0	0%		6	10.2%
2		16	28%		14	23.7%
3		25	43%		24	40.7%
4		15	26%		7	11.9%
5		2	4%		6	10.2%
* ≥ 3 (Frail)*		*42*	*72%*		*37*	*63%*
Cognitive impairment on ACE-R (score < 80)*	58	49	84%	59	35	60%
Diagnosis of:						
Parkinson’s disease	58	2	3.5%	56	2	3.5%
Depression	58	8	13.8%	58	7	12.1%
Heart Failure	58	12	20.7%	58	10	17.2%
COPD	58	11	19.0%	58	7	12.1%
Hip fracture	58	2	3.5%	58	1	1.7%
Stroke	58	9	15.5%	58	11	19.0%
Hypertension	58	33	56.9%	58	36	62.1%
MI	58	4	6.9%	58	4	6.9%
OA	58	27	46.6%	58	28	48.3%
Other arthritis	58	4	6.9%	58	5	8.7%
Diabetes	58	20	34.5%	58	20	34.5%
Dementia	51	3	5.9%	51	5	9.8%
Measure	Usual care			Frame Fit Intervention		
	*N*	*Mean*	*SD*	*N*	*Mean*	*SD*
Demographics: Age*	58	84.9	6.6	59	81.9	8.6
Number of conditions	58	5.4	0.3	58	5.0	0.3
Number of medications	57	8.7	0.6	59	7.8	0.4
ACE-R	58	70.0	13.3	59	71.2	17.1
FESI	56	33.31	5.23	57	30.05	7.60
IPEQ	57	7.31	8.61	58	7.00	6.97
Balance score total in secs) #	58	21.67	8.95	59	21.63	8.68
Gait speed at baseline (m/sec)	58	0.21	0.15	59	0.20	0.14
Timed up and go (secs)	57	65.81	36.60	56	84.66	88.31
Steps to turn	57	7.37	8.24	56	6.59	3.30
Grip strength (kg)	57	16.39	5.74	59	18.37	6.33
Equation 5D score	58	0.51	0.28	59	0.45	0.29
	*Total*	*Median*	*IQR*	*Total*	*Median*	*IQR*
Timed up and go (secs)	57	61	38–84	56	62	40–93

*significant between group difference (*P* < 0.05)

#Balance score reflects time spent in increasingly difficulty balance positions (10 = able to stand in normal standing position for 10 s, 20 = able to stand with feet together for 10 s, 30 = able to near tandem stand for 10 s, 40 = able to tandem stand for 10 s and 50 = able to stand on one leg for 10 s

### Safety

Two intervention-related AEs were recorded; one participant had a fall while exercising and required a two day stay in hospital as a result, and another participant reported feeling unsteady on their feet two hours after doing the home exercises. This participant had been doing the exercises 2–3 times a day despite being advised to exercise 3 times a week and these symptoms resolved fully when the prescription was followed.

One hundred and ten adverse events (AEs) were recorded in the trial database for 50 participants. There were more recorded in the intervention arm *N* = 64 compared to usual care *N* = 46. There were high levels of emergency department attendances and hospital admission in both trial arms (see Table 4 for data on AEs and secondary care use).

**Table 4:** Adverse events and secondary healthcare use

	Total number in each trial arm						
	AEs (trial database)	ED attendances (electronic records)		Hospital admissions (electronic records)		Days as an inpatient (electronic records)	
		0–6 m	6–12 m	0–6 m	6–12 m	0–6 m	6–12 m
Intervention	64	33	21	24	28	346	301
Usual care	46	49	14	28	27	449	372

ED attendances: <24 h stay in emergency dept or clinical decision unit

Hospital admission: Admission to a hospital ward

Days as an inpatient: Total days in hospital

**Fig. 4:**
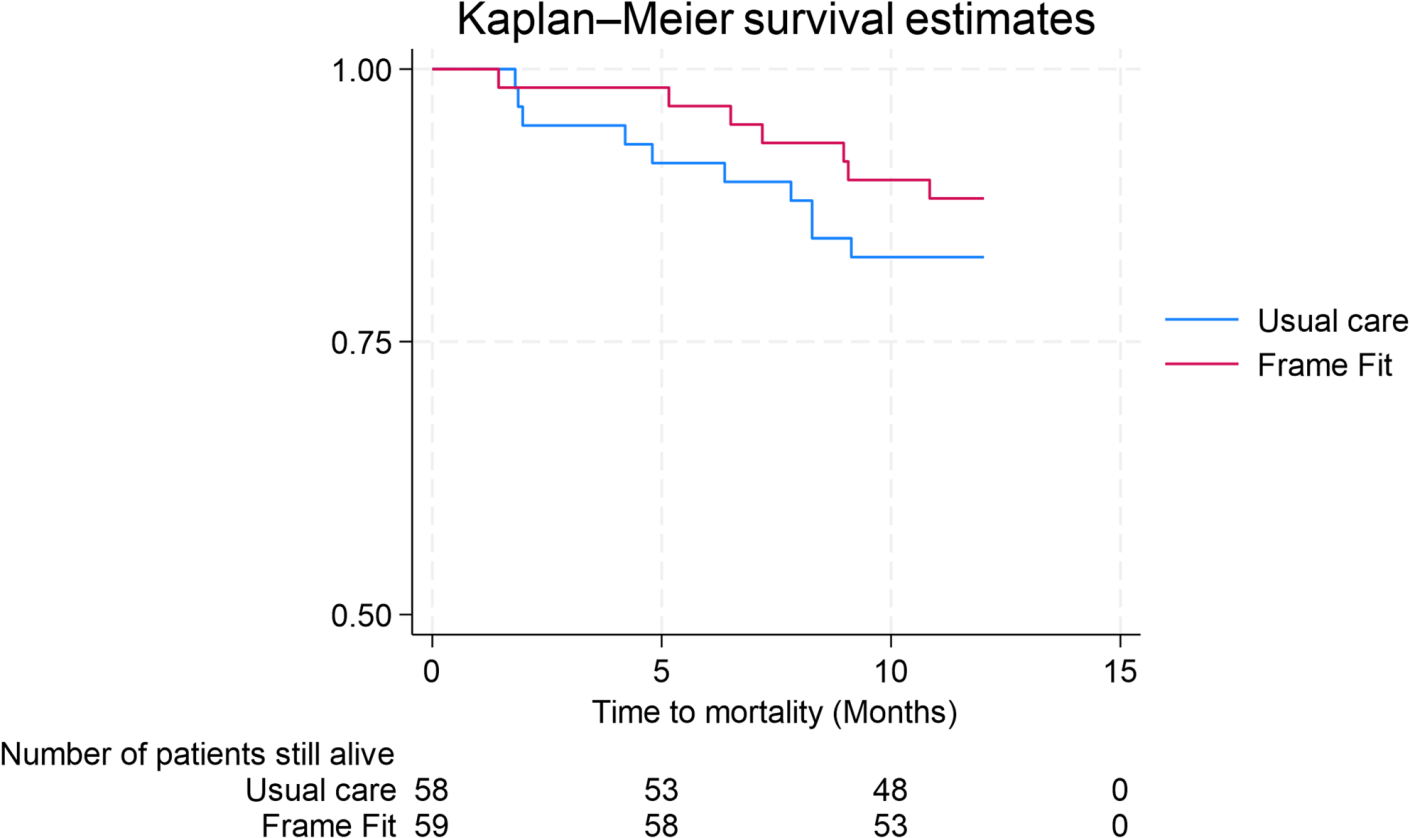
KM graph measured until 1 year after randomisation

In the 12 months after randomisation, 17 (15%) participants had died, 7 from the Frame Fit intervention and 10 from the usual care arm (Kaplan-Meier, Fig. 4). Cox regression analysis adjusted for age and sex indicated no significant between group difference in time to mortality: adjusted Hazard Ratio = 0.66, 95%CI 0.25–1.75.

### Acceptability and feasibility

Eighteen participants (31%) from intervention and 13 (22%) from the usual care arm dropped out during the intervention period (between baseline and 6-month follow up). Frame Fit intervention group participants completed a mean of 6.1 out of 8 possible sessions with the physiotherapist (76.7%) and the mean engagement score per patient over all sessions undertaken was 6.9 /10.

There were difficulties completing gait-related secondary outcome measures due to lack of adequate space or equipment. At baseline, it was not possible to perform a Timed Up and Go (TUG) correctly for 43 (37%) participants and lack of space precluded performing at all for four (3%). The TUG had to be shortened to less than 3 m for four participants, a change of direction (instead of walking straight ahead) was required for 11 participants and 34 participants had to start from unconventional seating such as a sofa, armchair or bed. Detailed notes were made of these adjustments so that follow up measurement could be performed in the same way. The mean distance walked to measure gait speed (excluding at least 0.5 m for acceleration and deceleration) was 2.4 m (range 1–6 m). Eleven (9%) participants had sufficient space to undergo gait speed testing over four or more metres.

### The intervention as prescribed

A mean of four out of a possible five warm up exercises were prescribed with no change between sessions. There was an increase in the number of strength exercises prescribed from a mean of two to a mean of three out of five exercises by the third session. A mean of one balance exercise was prescribed on the first session, only rising to four out of a possible 11 by the final session. Prescribed ankle weights started at a mean of 0.5 kg increasing to a mean of 1 kg. On the first session 58% were prescribed an ankle weight, increasing to 83% at session 8. The maximum weight used was 5.5 kg (see Table 5). Exercise diaries were available for 13 participants, of which four did not provide the full six months. The remaining 9 participants reported exercising 2.9 times per week.

**Table 5:** Exercise prescription

Type of exercise		Mean number of exercises prescribed per session						
		*N*	Mean	SD	Min	Max		
Warm up exercises (*N* = 5)	Session 1	52	4.5	0.7	3	5		
	Session 2	49	4.5	0.8	2	5		
	Session 3	47	4.6	0.7	3	5		
	Session 4	43	4.4	1.0	1	5		
	Session 5	43	4.4	1.0	1	5		
	Session 6	43	4.4	1.0	1	5		
	Session 7	36	4.2	1.2	1	5		
	Session 8	30	4.3	1.0	2	5		
Strength exercises (*N* = 5)	Session 1	52	2.6	1.6	0	5		
	Session 2	50	2.9	1.3	0	5		
	Session 3	47	3.4	1.2	0	5		
	Session 4	45	3.4	1.4	0	5		
	Session 5	46	3.5	1.3	0	5		
	Session 6	44	3.5	1.4	0	5		
	Session 7	38	3.3	1.6	0	5		
	Session 8	31	3.6	1.5	0	5		
Balance exercises (*N* = 11)	Session 1	52	1.3	1.5	0	7		
	Session 2	50	2.5	1.9	0	7		
	Session 3	47	3.5	2.0	0	8		
	Session 4	45	4.2	2.1	0	9		
	Session 5	46	4.3	2.3	0	9		
	Session 6	44	4.3	2.4	0	9		
	Session 7	38	4.0	2.5	0	9		
	Session 8	31	4.3	2.5	0	9		
							No weight prescribed (N)	No weight prescribed (%)
Ankle weight prescribed	Session 1	45	0.6	0.6	0	2	19	42.2
	Session 2	44	0.8	0.5	0	2	9	20.5
	Session 3	45	1.0	0.9	0	5.5	7	15.6
	Session 4	40	1.0	0.6	0	3	5	12.5
	Session 5	44	0.9	0.7	0	3	10	22.7
	Session 6	38	1.0	0.6	0	2	5	13.2
	Session 7	35	1.0	0.8	0	3	10	28.6
	Session 8	24	1.3	0.9	0	4	4	16.7

### Clinical effectiveness

#### Falls

During the first 6 months of follow up, 20 (35%) participants in the usual care group had 24 falls and 25 (42%) participants in the intervention group had 52 falls. Risk of being a faller was the same for each arm (Risk ratio 1.23, 95%CI 0.77–1.95) and rates of falls were higher in the intervention arm: IRR adjusted for age and sex: 2.10, 95%CI 1.06–3.98 and unadjusted IRR: 2.13, 95%CI 1.12–4.07). Over the 12 month follow up, 25 (45%) participants in the usual care group had 43 falls (minimum = 0, maximum = 5, IQR = 0–2) and 26 (44%) participants in the Frame Fit intervention group had 81 falls (minimum = 0, maximum = 23, IQR = 0–1). Risk of being a faller was the same for each arm (Risk ratio 0.98, 95%CI 0.66–1.74) and there was a trend towards higher rates of falls in the intervention arm: IRR adjusted for age and sex: 1.76, 95%CI 0.93–3.33 and unadjusted IRR: 1.82, 95%CI 0.98–3.39 (see Table 6 for falls data).

**Table 6:** Details of falls

Number of falls		Usual care *N* = 58	Frame Fit Intervention *N* = 59	
	6 months	24	52	
Number of fallers	6 months	20	25	
Number of falls	12 months	43	81	
Max falls per person	12 months	5	23	
IQR	12 months	0–2	0–1	
Number of fallers	12 months	25	26	
Falls rate (per person per year)	12 months	0.74	1.37	
Adjusted incidence rate ratio	6 months	2.10 (95%CI 1.06–3.98)		
Adjusted incidence rate ratio	12 months	1.76 (95%CI 0.93–3.33)		
Risk ratio (any fall)	6 months	1.23 (95%CI 0.77–1.95)		
Risk ratio (any fall)	12 months	0.98 (95%CI 0.66–1.74)		
		Usual care *N* = 12		Frame Fit Intervention *N* = 19
Ratio of unit of activity (IPEQ-FU) /per fall (at 6 months) analysed in fallers only.	6 months	6.19 (SD 8.15)		4.62 (SD 3.83)

#### Secondary outcomes

There were no between group differences identified for any of the secondary outcome measures collected - see Table 7 and no difference in proportion who improved in balance and gait measures (Table 8). The IPEQ per fall calculation was only possible for those with at least one fall in the 6 month follow up and due to missing data numbers were too small for meaningful analysis with 12 observations in the usual care and 19 in the frame fit intervention group.

**Table 7:** Change in secondary outcome measures between baseline and follow-up

Measure	Usual care		Frame Fit intervention		
	*N*	Mean at follow up (SD)	*N*	Mean at follow up (SD)	Adjusted OR (95% CI)
FESI	50	33.9 (6.6)	41	32.2 (6.9)	-0.32 (-3.05, 2.41)
IPEQ	50	5.8 (8.5)	40	4.7 (4.3)	-1.68 (-4.54, 1.19)
Balance score	54	12.7 (12.7)	47	15.5 (15.2)	3.10 (-2.35, 8.55)
Gait speed	42	0.2 (0.3)	35	0.2 (0.2)	-0.16 (-0.11, 0.80)
Timed up and go	44	163.8 (168.4)	33	132.0 (142.7)	-32.31 (-102.62, 37.99)
Grip strength	46	14.5 (6.9)	34	15.9 (7.5)	-0.03 (-2.72, 2.66)
Equation 5D	51	0.4 (0.4)	42	0.4 (0.4)	0.11 (-0.14, 0.17)

Adjusted for age, sex and baseline score

**Table 8:** Improvement categories for balance and Timed up and Go

	**Usual care ** **(N)**		**Frame Fit intervention ** **(N)**	
Change in balance category:				
Reduction in performance by 4	1		0	
Reduction in performance by 3	4		0	
Reduction in performance by 2	9		9	
Reduction in performance by 1	10		8	
No change in performance	13		14	
Improvement in performance by 1	8		9	
Improvement in performance by 2	1		2	
Improvement in performance by 3	1		0	
Any improvement in balance performance	10		11	
Timed up and go category TUAG < 30 s	Baseline	Follow up	Baseline	Follow up
	5	7	9	5
Change in TUAG category	3		2	
Improvement in performance (into < 30 s cat)				
Reduction in performance (into > 30 s cat)	7		0	
No change in performance	48		57	

## Discussion

This randomised controlled trial was particularly challenging to recruit to. We enrolled 117 participants and did not achieve the required sample size to be powered to detect a difference in fall rates. Recruitment was affected by high numbers who did not wish to participate (*n* = 233 + 12) and almost as many who were not eligible for the study (*n* = 202 + 5).

The Frame Fit intervention offered a comparable safety profile to other fall prevention exercise interventions [17], with two reported intervention-related adverse events. While there were more dropouts in the intervention group, adherence to physiotherapy sessions was good, participants demonstrated good engagement and for the small number who kept exercise diaries, they reported doing the home exercises the required number of times per week. While most of the available warm up and strength exercises were prescribed, on average, fewer than half of the available balance exercises were added to the programme and the average level of ankle weight prescribed was low (< 2 kg) – the original OEP studies the reported ranges of weights between 0 and 6 kg [34].

Those in the Frame Fit intervention arm fell more in the first 6 months of follow up, but the difference between groups was not significant at 12 months. There was a trend (as observed in the Kaplan Meier graph) of lower mortality in the Frame Fit intervention group, but this was not statistically significant. Balance scores appeared to improve more in the intervention group, but this was not statistically significant due to large confidence intervals and there was no evidence of effect on any other secondary outcomes at 6-month follow up. Due to the study being underpowered for the primary outcome and key secondary (explanatory) outcomes, caution should be taken when interpreting these findings.

The wider literature on the effect of exercise on falls is mixed and while synthesis of exercise studies indicates effectiveness, there is heterogeneity in these findings [17]. Original OEP trials conducted in New Zealand, upon which Frame Fit was modelled, demonstrated effectiveness in reducing falls [34]. However, a large pragmatic trial undertaken in primary care in the UK (the PreFIT study) found that while there were small effects on quality of life, the OEP had no significant effect on falls [36]. Another study, again conducted in the UK, (ProACT65+) compared group-based exercise to OEP and usual care and only group exercise reduced fall risk [37]. Recent trials have explored various adaptations of the OEP and found it effectively reduced falls when delivered with more frequent scheduled home visits than the original programme [38], when combined with a weekly outpatient clinic visits [39] or when delivered via telerehabilitation [40]. However, delivery in group-based workshops did not reduce risk of falls [41].

It is unclear whether there are differences in the outcomes from these trials of the same intervention are due to variations in characteristics associated with the intervention or the case mix of study cohorts. Group-based exercise may support better adherence and enable participants to engage in more challenging exercise resulting in a better dose of the intervention. The Frame Fit study cohort were physically frail, nearly three quarters scored ≥ 3 on the Fried Frailty Index, their average standing balance was poor, and gait speed was extremely slow. In addition, the mean ACE-R score indicated most participants were living with a degree of cognitive impairment [22]. Evidence suggests that without sufficient dose of exercise, the effect on falls is attenuated [42]. Performing highly challenging balance exercise in an unsupervised home setting, for individuals with poor baseline balance function and impaired cognition could increase the risk of falls while exercising leading prescribing physiotherapists to show caution in the degree of challenge offered. There was evident caution in prescribing balance exercises. However, there was only one recorded fall that occurred while doing the Frame Fit exercises. While there is a substantial evidence base for fall prevention exercise, the impact of frailty on effectiveness is not clear. A review of the inclusion criteria for all studies included in the Sherrington Cochrane review [17] and the update undertaken for NICE guideline production [43] found only five studies that explicitly recruited based on frailty status [44–48], four of which sought to recruit a cohort living with frailty [44, 46–48] and of these, only one found a reduction in falls [47].

Frailty is associated with symptoms of exhaustion, which may be a barrier to exercise participation, particularly for home exercise programmes where self-motivation is required. Older people living with frailty will likely need to make greater gains in muscle strength and balance skill through exercise to see an effect on falls. This creates a ‘double disadvantage’ in that more exercise is required but attaining effective doses is less achievable. Additionally, there were high numbers of emergency department attendance and hospital admissions in both arms during the 6 months intervention period. Exacerbation of comorbidities would impact on the ability to maintain a home exercise programme as exercises would need to be regressed following a hiatus in participation due to illness. The control group fall rate in this study (0.74 falls per person per year) is slightly lower than median rate calculated by Wang et al., in a systematic review investigating the relative effectiveness of exercise based on control group fall rate [49]. Wang’s review identified a trend towards exercise being more effective in cohorts with control group fall rates higher than the median. It is possible that this cohort of older people in the Frame Fit study, living with frailty and substantial mobility impairments, had a lower baseline fall rate due to lower levels of activity and exposure to falls. Introducing an exercise intervention that does not meet the necessary intensity and dose risks increasing exposure to falls without the adequate acquisition of improved physical performance to withstand the increased threats to balance of being more mobile. A brisk walking intervention for post-menopausal women increased falls [50] as did an exercise programme for older people immediately after hospital discharge [51]. The increase in falls in the Frame Fit intervention arm during the 6-month intervention period supports this hypothesis.

There is evidence from other research that exercise is feasible and can slow the progress of, and even reverse frailty in older people [13]. However, to prevent falls in people living with frailty, interventions need to address the multi-factorial nature of risk factors present. Frame fit participants had multiple long-term conditions and were taking multiple medications suggesting that exercise as a single intervention, even if it resulted on physiological changes, would not be sufficient to affect fall risk. In fact, recent national [43] and international guidelines [52] recommend multifactorial or comprehensive approaches for older people living with frailty. Given the inherent risks associated with walking aid use described in the introduction, interventions that include environmental assessment and modification may yield the most benefit in this population [53]. Furthermore, work to develop novel walking frames that provide better support and manoeuvrability may be another avenue to address fall risk [54].

There were several limitations to this study. Firstly, the data collection for this study was completed 6 years ago. Secondly, despite designing a pragmatic approach to identify as many eligible participants as possible, it was not possible to reach the recruitment target. The study was underpowered due to significant challenges with recruitment. Only 21% of those approached were randomised with more than one third of those approached declining to participate and another third being ineligible. This suggests the intervention was not appealing to this population and it would only be applicable to a minority of patients. Both these factors would limit effective implementation, had the findings indicated this. Furthermore, where reasons for declining participation were provided, reluctance to be in a trial was more frequently given than not wishing to exercise, perhaps reflecting apprehension around research participation in this population.

Reporting of AEs and falls could have been biased towards greater reporting for intervention participants, due to the regular contact with the physiotherapists. Indeed, rates of falls were higher in the intervention group during the first 6 months, when visits would have been taking place. While more AEs were reported in the intervention arm, emergency department attendance and hospital admission data collected from healthcare records were not higher. Alternative methods to measure fall rates are required in trials where participants may not reliably report all falls, for example the use of wearable technology or only measuring falls recorded in healthcare records. Data on evaluation of unblinding at follow up was poorly collected but where it was recorded, this occurred frequently.

The evidence from this study does not support further investigation into the Frame Fit intervention as a standalone fall prevention intervention. However, it may be considered as the exercise component of a comprehensive intervention addressing multiple fall risk factors for people with frailty. An example may be combining Frame Fit exercises with Comprehensive Geriatric Assessment, home hazard assessment and modification, additional behavioural approaches around appraising fall risk and development of biomechanically enhanced walking frames. More research is needed to better understand how to prevent falls in this population as no other studies have specifically focused on exercise for walking frame users.

## Conclusion

The Frame Fit intervention was safe and feasible to deliver for the small proportion of those willing and eligible to participate. As uptake was low, it was underpowered to detect a difference in the primary outcome of falls. No significant differences were identified in falls rates at 12 months or any of the secondary outcome measures. The prevalence of frailty and high frequency emergency department attendance and hospital admission may have limited the impact of the exercise programme.

## Electronic Supplementary Material

Supplementary Material 1.

## Data Availability

The datasets generated and/or analysed during the current study are not publicly available as this was not included in the original ethics approval but are available from the corresponding author on reasonable request.
